# Ibrutinib and rituximab plus cyclophosphamide, doxorubicin, vincristine and prednisone in patients with previously untreated non‐germinal centre B‐cell‐like diffuse large B‐cell lymphoma: A Chinese subgroup analysis of the phase III PHOENIX trial

**DOI:** 10.1002/jha2.517

**Published:** 2022-08-30

**Authors:** Jun Zhu, Xiaonan Hong, Yu Qin Song, Brendan Hodkinson, Sriram Balasubramanian, Songbai Wang, Qingyuan Zhang, Yuankai Shi, Huiqiang Huang, Huilai Zhang, Yan Zhu, Stephen Martin Shreeve, Steven Sun, Ze Wang, Xiaocan Wang, Yue Fan, Wyndham Wilson, Jessica Vermeulen

**Affiliations:** ^1^ Beijing University Cancer Hospital and Institute Beijing China; ^2^ Fudan University Cancer Hospital Shanghai China; ^3^ Oncology Translational Research Janssen Research and Development Spring House Pennsylvania USA; ^4^ Oncology Translational Research Janssen Research and Development San Diego California USA; ^5^ Oncology Translational Research Janssen Research and Development Raritan New Jersey USA; ^6^ Affiliated Cancer Hospital of Harbin Medical University Harbin China; ^7^ National Cancer Center/National Clinical Research Center for Cancer/Cancer Hospital Chinese Academy of Medical Sciences and Peking Union Medical College Beijing Key Laboratory of Clinical Study on Anticancer Molecular Targeted Drugs Beijing China; ^8^ Sun Yat‐sen University Cancer Center Guangzhou China; ^9^ Tianjin Cancer Hospital Heping China; ^10^ Janssen Research and Development Raritan New Jersey USA; ^11^ Clinical Oncology Janssen Research and Development San Diego California USA; ^12^ Clinical Biostats Janssen Research and Development Raritan New Jersey USA; ^13^ Xian Janssen Pharmaceuticals Medical Affairs Beijing China; ^14^ Oncology Translational Research Janssen Research and Development Shanghai China; ^15^ National Cancer Institute National Institutes of Health Bethesda Maryland USA; ^16^ Clinical Oncology Janssen Research and Development Leiden The Netherlands

**Keywords:** China, DLBCL, ibrutinib, previously untreated

## Abstract

In this post hoc subgroup analysis of 200 patients enrolled in China from the phase III PHOENIX trial (*N *= 838, NCT01855750), addition of ibrutinib to rituximab plus cyclophosphamide, doxorubicin, vincristine and prednisone (R‐CHOP) did not improve event‐free survival (EFS) versus placebo+R‐CHOP in the intent‐to‐treat (ITT; *n* = 200, hazard ratio [HR] = 0.83, 95% confidence interval [CI]: 0·509–1.349; *p *= 0.4495) or activated B‐cell‐like (ABC; *n* = 141 [based on available gene‐expression profiling data], HR = 0.86, 95% CI: 0.467–1.570; *p *= 0.6160) subpopulations. However, ibrutinib+R‐CHOP improved EFS (HR = 0·50, 95% CI: 0.251–1.003) and progression‐free survival (PFS; HR = 0.48, 95% CI: 0.228–1.009) versus placebo+R‐CHOP in patients aged <60 but not ≥60 years. Grade ≥3 serious treatment‐emergent adverse events occurred more with ibrutinib+R‐CHOP (45·6% vs. 31·3%). The percentage of patients receiving ≥6 cycles of R‐CHOP was similar across treatment arms in those <60 years. A numerical trend was seen towards improved EFS and PFS with ibrutinib+R‐CHOP versus placebo+R‐CHOP in patients with *MYC*‐high/*BCL2*‐high co‐expression. In this slightly younger Chinese subgroup, ibrutinib+R‐CHOP did not improve EFS in the ITT and ABC subpopulations but improved outcomes with manageable safety in patients <60 years, consistent with overall PHOENIX study outcomes.

## 1 INTRODUCTION

Over the last decade, prevalence of lymphoma in China has continued to rise, with non‐Hodgkin lymphoma (NHL) accounting for 68,500 new cases and an estimated 37,600 cancer‐related deaths in 2016 [1, 2]. Diffuse large B‐cell lymphoma (DLBCL) accounts for up to 40% of lymphoma worldwide [3] and up to 50% of all newly diagnosed NHL in China [4]. The non‐germinal centre B‐cell‐like (non‐GCB) subtype of DLBCL is more commonly seen in Chinese patients. In a study comparing 124 patients with DLBCL from China versus 114 patients from the United States and Western Europe, non‐GCB subtype was confirmed in 78.2% and 72.6% of patients in China by Hans and Choi algorithm, and 47.4% and 43.9% in the Western cohort, respectively [5].

Rituximab, cyclophosphamide, doxorubicin, vincristine and prednisone (R‐CHOP) is the standard first‐line treatment for patients with DLBCL [2, 6, 7]. The improved efficacy of R‐CHOP versus CHOP has been established in Chinese patients [8, 9]. In a randomised study of DLBCL in China (*N *= 63), first‐line R‐CHOP provided a significantly lower rate of disease progression versus CHOP (3.2% vs. 21.9%; *p *= 0.026), with slightly higher overall (objective response rate [ORR]; 83.8 vs. 65.6%) and complete response (CR) rates (41.9% vs. 37.5%) [10]. In another study of 411 patients with previously untreated DLBCL, R‐CHOP significantly improved ORR (95.19% vs. 87.95%, *p *= 0·007), CR (77.01% vs. 71.43%), progression‐free survival (PFS; *P *= 0·018) and overall survival (OS; *p *= 0.034) [11]. Real‐world analyses for first‐line R‐CHOP in Chinese patients with DLBCL have shown CR rates of 55%–77%, 3‐year PFS/event‐free survival (EFS) rates of 59%–75% and 3‐year OS rates of 76%–90% [12, 13]. However, consistent with data in Western patients [14], high *MYC*/*BCL2* co‐expression, which has been observed in 18·4% of patients in China, has been associated with inferior survival in R‐CHOP‐treated Chinese patients with DLBCL [15, 16].

Ibrutinib is a first‐in‐class oral covalent Bruton's tyrosine kinase inhibitor, approved in China as monotherapy for patients with chronic lymphocytic leukaemia/small lymphocytic lymphoma, relapsed/refractory mantle cell lymphoma and relapsed/refractory Waldenström macroglobulinaemia or those unfit for first‐line chemoimmunotherapy [17]. The phase III PHOENIX study (NCT01855750) evaluated the outcomes of ibrutinib+R‐CHOP versus placebo+R‐CHOP in patients (*N *= 838) with untreated non‐GCB DLBCL [18]. The study did not meet its primary endpoint of EFS in the overall intent‐to‐treat (ITT; hazard ratio [HR] = 0.934, 95% confidence interval [CI]: 0.726–1.200) or activated B‐cell‐like (ABC) subpopulation (HR = 0.949, 95% CI: 0.704–1.279). However, a significant interaction between treatment and age was identified in a pre‐planned analysis. In the exploratory analysis, improvements were seen among patients aged <60 years in EFS (HR = 0.579, 95% CI: 0.380–0.881), PFS (HR = 0.556, 95% CI: 0.359–0.860) and OS (HR = 0.330, 95% CI: 0.162–0.670). Ibrutinib+R‐CHOP was associated with increased toxicity in patients aged ≥60 years. In patients aged <60 years, any‐grade adverse events (AEs) and grade ≥3 AEs were similar between arms; while serious AEs and AEs leading to R‐CHOP discontinuation were higher in the ibrutinib+R‐CHOP arm, safety was manageable. The current analysis aimed to evaluate outcomes of patients enrolled from China in the PHOENIX study.

## 2 METHODS

### 2.1 Study design, treatment and assessment

PHOENIX was a randomised, double‐blind, phase III, international study conducted in 28 countries across North America, Europe, Asia, Latin America and Australia. The methodology for the PHOENIX study has been reported previously [18].

Briefly, patients with untreated non‐GCB DLBCL were randomized 1:1 to receive either oral ibrutinib (560 mg/d) or placebo, in addition to R‐CHOP (pre‐specified six or eight cycles). Randomisation was stratified by Revised International Prognostic Index, region and number of pre‐specified treatment cycles (six vs. eight cycles).

The primary endpoint was investigator‐assessed EFS in the ITT population and ABC subpopulation. EFS was defined as time from randomization to disease progression, relapse after CR, initiation of subsequent disease‐specific therapy for positron emission tomography–positive or biopsy‐proven residual disease after ≥6 cycles of R‐CHOP or any‐cause death.

Secondary study endpoints included PFS, OS, CR rates and safety. Duration of response (DOR) based on EFS events was estimated for patients who achieved CR or partial response. Exploratory analyses included outcomes in patients aged <60 years versus ≥60 years.

Concordance between immunohistochemistry and gene‐expression profiling (GEP) classification of molecular subtype was determined using matching tumour or blood samples. Pre‐planned biomarker analyses included evaluation of *BCL2* and *MYC* gene expression by RNA‐seq and whole exome sequencing (details in Supporting Information).

### 2.2 Statistical analysis

EFS, PFS and OS were compared between arms using stratified log‐rank test and Cox proportional hazards model. Survival distribution was estimated using the Kaplan‐Meier product‐limit method. CR rates were compared using the Cochran‐Mantel‐Haenszel X^2^ test (relative risk) and logistic regression analysis (odds ratio), adjusted for stratification factors. Biomarker analysis details are included in the Supporting Information.

The analysis in the Chinese subgroup was post hoc. All *p* values are nominal.

## 3 RESULTS

### 3.1 Patients

**FIGURE 1:**
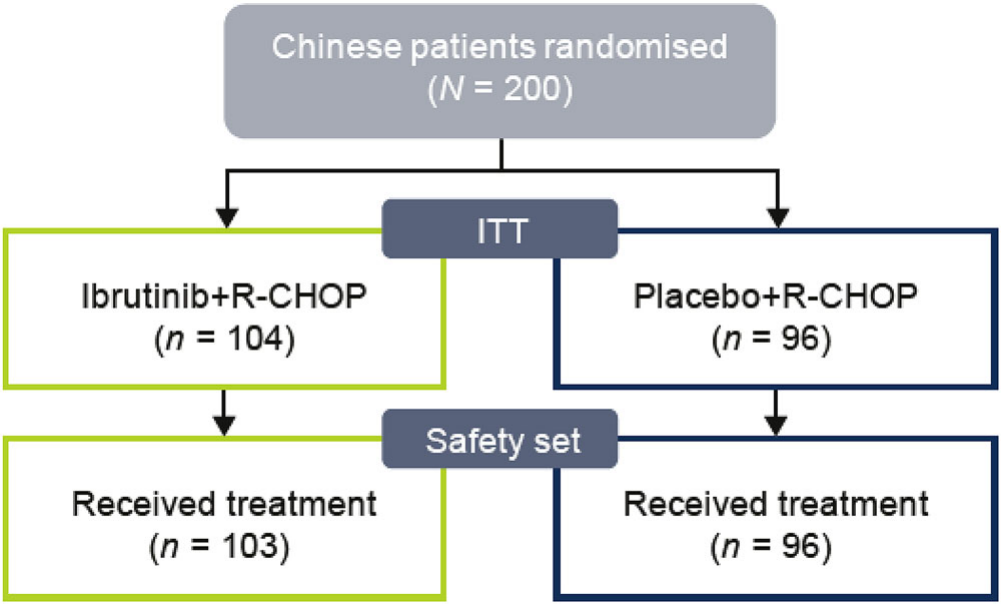
Patient disposition. ITT, intent‐to‐treat; R‐CHOP, rituximab plus cyclophosphamide, doxorubicin, vincristine and prednisone

In total, 200 patients were enrolled in the study from China; 104 received ibrutinib+R‐CHOP, and 96 received placebo+R‐CHOP (Figure 1). Here, we report results in this Chinese subgroup.

Baseline characteristics were well balanced between treatment arms (Table 1), with median age 59.0 years and 53% of patients aged <60 years in both arms; 54.8% of patients in the ibrutinib+R‐CHOP and 60.4% in the placebo+R‐CHOP group were male. More patients in the placebo+R‐CHOP arm had an Eastern Cooperative Oncology Group performance status of 2 than in the ibrutinib+R‐CHOP arm (24% vs. 12·5%). Median (95% CI) time on the study was 32.6 (31.4–33.6) months.

**TABLE 1:** Baseline characteristics of Chinese patients in the phase III PHOENIX study

	Ibrutinib+R‐CHOP	Placebo+R‐CHOP
	*n* = 104	*n *= 96
Age (years)		
Mean (SD)	57·6 (10·26)	55·1 (14·22)
Median (range)	59·0 (25–77)	59·0 (19–80)
Age groups, *n* (%)		
<60 years	55 (52·9)	51 (53·1)
≥60 years	49 (47·1)	45 (46·9)
Sex, *n* (%)		
Female	47 (45·2)	38 (39·6)
Male	57 (54·8)	58 (60·4)
Time from initial diagnosis to randomisation, days		
Mean (SD)	26·9 (30·45)	30·1 (36·35)
Median (range)	20·0 (6–302)	21·0 (9–349)
Baseline stage of DLBCL at entry, *n* (%)		
I	0	0
II	35 (33·7)	38 (39·6)
III	32 (30·8)	26 (27·1)
IV	37 (35·6)	32 (33·3)
Baseline lymphoma symptoms, *n* (%)	20 (19·2)	21 (21·9)
Bone marrow involvement, *n* (%)*	5 (4·8)	3 (3·1)
ECOG PS, *n* (%)		
0	42 (40·4)	36 (37·5)
1	49 (47·1)	37 (38·5)
2	13 (12·5)	23 (24·0)
Bulky tumour (long axis ≥10 cm), *n* (%)	7 (6·7)	10 (10·4)
Number of extranodal sites, *n* (%)		
0	37 (35·6)	32 (33·3)
1	39 (37·5)	36 (37·5)
> 1	28 (26·9)	28 (29·2)
IPI/R‐IPI score index number, *n* (%)		
0	0	0
1	35 (33·7)	36 (37·5)
2	33 (31·7)	31 (32·3)
3	31 (29·8)	20 (20·8)
4	5 (4·8)	7 (7·3)
5	0	2 (2·1)
Elevated LDH, n (%)	56 (53·8)	40 (41·7)
Number of planned treatment cycles (used in stratification), *n* (%)		
6	17 (16·3)	25 (26·0)
8	87 (83·7)	71 (74·0)
GEP^†^ subtype, *n* (%)	94	84
ABC	72 (76·6)	69 (82·1)
Unclassified	7 (7·4)	2 (2·4)
GCB	15 (16·0)	13 (15·5)

Abbreviations: ABC, activated B cell‐like; DLBCL, diffuse large B‐cell lymphoma; ECOG PS, Eastern Cooperative Oncology Group performance status; GCB, germinal centre B cell‐like; GEP, gene expression profiling; IPI, International Prognostic Index; LDH, lactate dehydrogenase; R‐CHOP, rituximab plus cyclophosphamide, doxorubicin, vincristine and prednisone; R‐IPI, revised International Prognostic Index.

* Defined as any baseline aspirate or biopsy result of histology positive or histology negative/intermediate that is confirmed positive by immunohistochemistry or flow cytometry.

^†^ Conducted after non‐GCB enrichment by immunohistochemistry. Samples were evaluable in 94 patients who received ibrutinib+R‐CHOP and 84 patients who received placebo+R‐CHOP. Patients with missing samples (ibrutinib+R‐CHOP, *n* = 2; placebo+R‐CHOP, *n* = 2) or test failure (ibrutinib+R‐CHOP, *n* = 8; placebo+R‐CHOP, *n* = 10) were not included in the analysis.

Valid GEP data were available for 178/200 patients (Table 1). Of these patients, 79.2% (141/178) had ABC subtype, 15.7% (28/178) had GCB, and 5.1% (9/178) were unclassified. The concordance of GEP with immunohistochemistry for patients with non‐GCB subtype was 84.3% (150/178).

### 3.2 Efficacy

**FIGURE 2:**
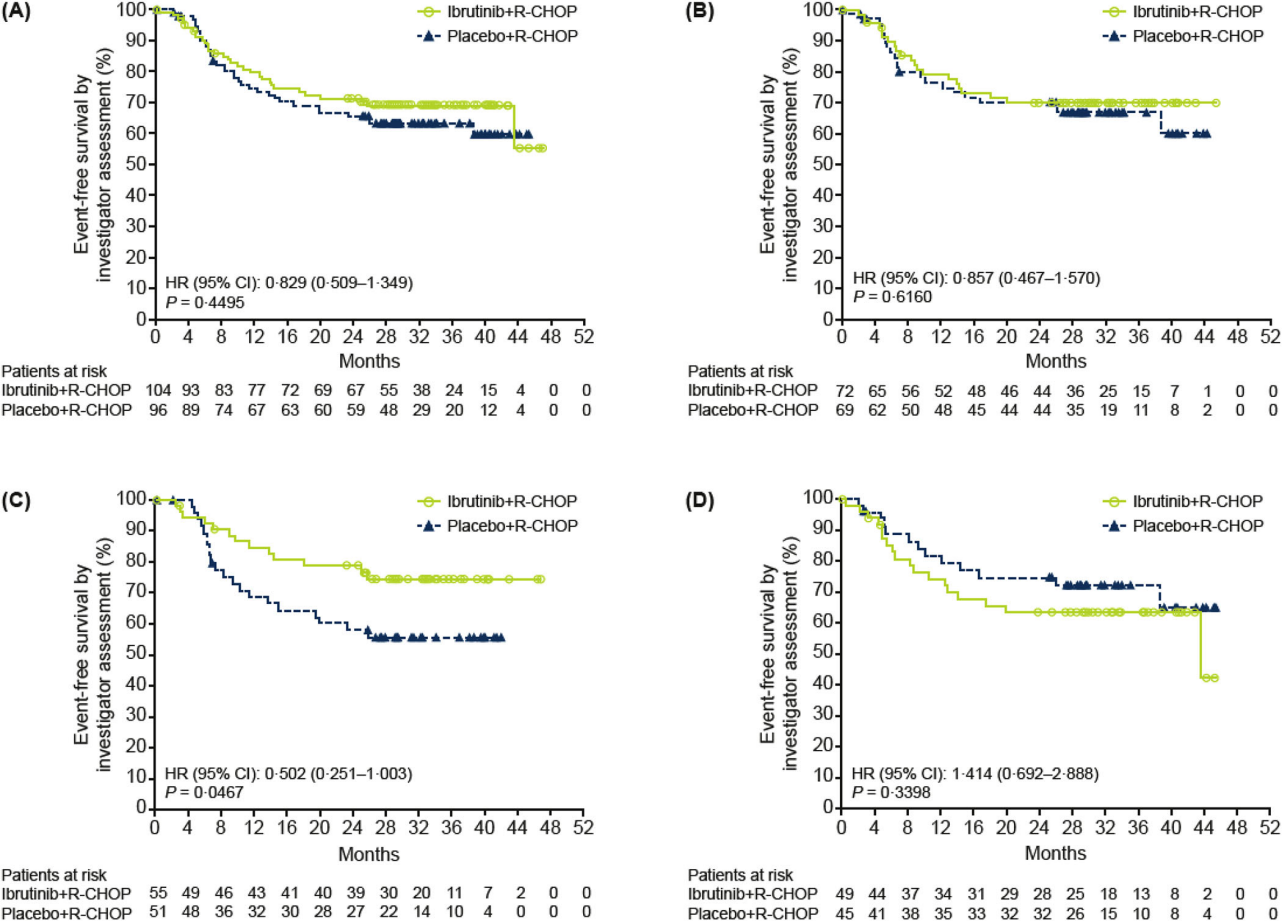
Kaplan‐Meier survival curves for event‐free survival. (A) ITT population—China subgroup; (B) ABC population—China subgroup; (C) ITT population—China subgroup, patients aged <60 years; (D) ITT population—China subgroup, patients aged ≥60 years. CI, confidence interval; HR, hazard ratio; ITT, intent‐to‐treat; R‐CHOP, rituximab plus cyclophosphamide, doxorubicin, vincristine and prednisone

**FIGURE 3:**
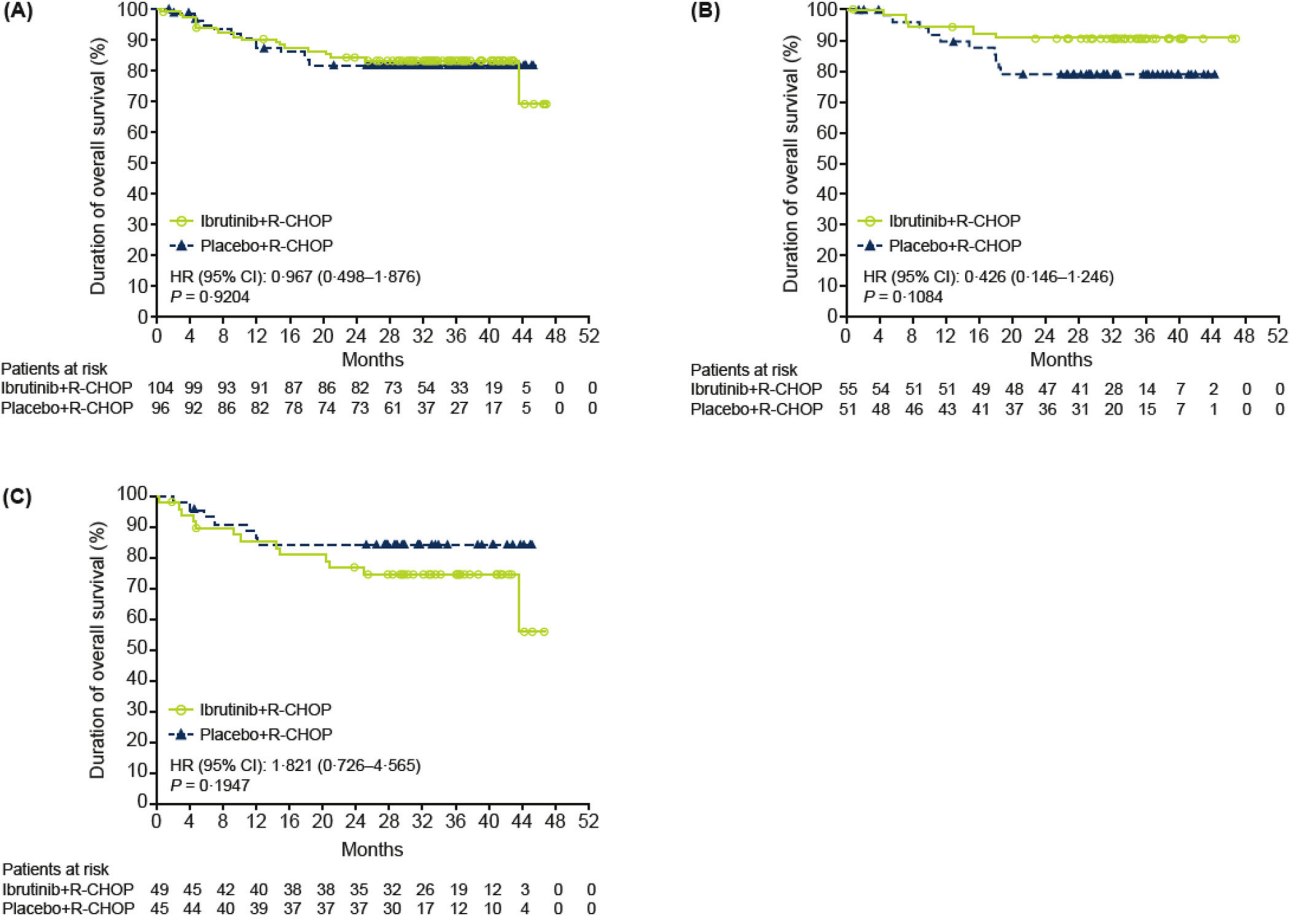
Kaplan‐Meier survival curves for overall survival. (A) ITT population—China subgroup; (B) ITT population—China subgroup, patients aged <60 years; (C) ITT population—China subgroup, patients aged ≥60 years. CI, confidence interval; HR, hazard ratio; ITT, intent‐to‐treat; R‐CHOP, rituximab plus cyclophosphamide, doxorubicin, vincristine and prednisone

The addition of ibrutinib to R‐CHOP did not improve EFS in the ITT population (HR = 0.83, 95% CI: 0.509–1.349; *p *= 0.4495; Table 2, Figure 2A) or ABC subpopulation (HR = 0.86, 95% CI: 0.467–1.570; *P *= 0.6160; Online Table SI, Figure 2B). Compared with placebo+R‐CHOP, ibrutinib+R‐CHOP did not improve PFS in the ITT population (HR = 0.83, 95% CI: 0.502–1.372; *p *= 0.4670; Table 2) or the ABC population (HR = 0.76, 95% CI: 0.410–1.425; *p *= 0.3958) or OS in the ITT population (HR = 0.97, 95% CI: 0.498–1.876; *p *= 0.9204; Table 2, Figure 3A) or the ABC population (HR = 0.93, 95% CI: 0.430–2.002; *p *= 0.8488).

**TABLE 2:** Efficacy of ibrutinib+R‐CHOP versus placebo+R‐CHOP among Chinese patients with DLBCL

	Age <60 years		Age ≥60 years		Total (ITT population – China subgroup)	
	Ibrutinib+R‐CHOP	Placebo+R‐CHOP	Ibrutinib+R‐CHOP	Placebo+R‐CHOP	Ibrutinib+R‐CHOP	Placebo+R‐CHOP
	*n* = 55	*n* = 51	*n* = 49	*n* = 45	*n* = 104	*n* = 96
**EFS**						
Number of events, *n* (%)	13 (23·6)	21 (41·2)	18 (36·7)	13 (28·9)	31 (29·8)	34 (35·4)
HR (95% CI) *p* value	0·502 (0·251–1·003) 0·0476		1·414 (0·692–2·888) 0·3398		0·829 (0·509–1·349) 0·4495	
Median (95% CI), months	NE (NE–NE)	NE (14·98–NE)	43·56 (17·48–NE)	NE (38·67–NE)	NE (43·56–NE)	NE (38·67–NE)
36‐month EFS rate (95% CI)	0·743 (0·597–0·842)	0·555 (0·403–0.684)	0·633 (0·477–0·753)	0·721 (0·561–0·831)	0·691 (0·589–0·773)	0·634 (0·525–0·724)
**PFS**						
Number of events, *n* (%)	11 (20)	19 (37·3)	18 (36·7)	13 (28·9)	29 (27·9)	32 (33·3)
HR (95% CI) *p* value	0·480 (0·228–1·009) 0·0476		1·414 (0·692–2·888) 0·3398		0·830 (0·502–1·372) 0·4670	
Median (95% CI), months	NE (NE–NE)	NE (19·81–NE)	43.56 (17·48–NE)	NE (38·67–NE)	NE (43·56–NE)	NE (38·67–NE)
36‐month PFS rate (95% CI)	0·782 (0·640–0·873)	0·598 (0·445–0·722)	0·633 (0·477–0·753)	0·721 (0·561–0·831)	0·712 (0·611–0·792)	0·56 (0·548–0·744)
**OS**						
Number of events, *n* (%)	5 (9·1)	10 (19·6)	13 (26·5)	7 (15·6)	18 (17·3)	17 (17·7)
HR (95% CI) *p* value	0·426 (0·146–1·246) 0·1084		1·821 (0·726–4·565) 0·1947		0·967 (0·498–1·876) 0·9204	
Median (95% CI), months	NE (NE–NE)	NE (NE–NE)	NE (43·56–NE)	NE (NE–NE)	NE (43·56–NE)	NE (NE–NE)
36‐month OS rate (95% CI)	0·907 (0·790–0·960)	0·789 (0·643–0·881)	0·746 (0·596–0·847)	0·842 (0·696–0·921)	0·831 (0·743–0·892)	0·815 (0·719–0·881)
**Best response**						
Overall response, *n* (%)	50 (90·0)	45 (88·2)	41 (83·7)	43 (95·6)	91 (87·5)	88 (91·7)
Complete response, *n* (%)	38 (69·1)	28 (54·9)	31 (63·3)	29 (64·4)	69 (66·3)	57 (59·4)
Partial response, *n* (%)	12 (21·8)	17 (33·3)	10 (20·4)	14 (31·1)	22 (21·2)	31 (32·3)
**Duration of response**						
Median, months (95% CI)	NE (NE–NE)	NE (42·78–NE)	45·90 (NE–NE)	NE (NE–NE)	NE (40·64–NE)	NE (NE–NE)
36‐month DOR rate (95% CI)	0·778 (0·693–0·842)	0·644 (0·557–0·717)	0·718 (0·653–0·774)	0·733 (0·663–0·791)	0·765 (0·660–0·842)	0·678 (0·566–0·767)

Abbreviations: CI, confidence interval; DLBCL, diffuse large B‐cell lymphoma; DOR, duration of response; EFS, event‐free survival; HR, hazard ratio; ITT, intent‐to‐treat; NE, not estimable; OS, overall survival; PFS, progression‐free survival; R‐CHOP, rituximab plus cyclophosphamide, doxorubicin, vincristine and prednisone.

### 3.3 Subgroup analysis by age

In patients aged <60 years in the ITT population, ibrutinib+R‐CHOP improved EFS (HR = 0.50, 95% CI: 0.251–1.003; *p *= 0.0476) and PFS (HR = 0.48, 95% CI: 0.228–1.009; *p *= 0.0476) versus placebo+R‐CHOP; a trend of improved OS was also observed (HR = 0.43; 95% CI: 0.146–1.246; *p *= 0.1084; Table 2, Figures 2C and 3B). The 36‐month EFS, PFS and OS were 74.3%, 78.2% and 90.7% with ibrutinib+R‐CHOP versus 55.5%, 59.8% and 78.9% with placebo+R‐CHOP, respectively. The ORR was similar between treatment arms (90.0% vs. 88.2%); however, CR rates were higher with ibrutinib+R‐CHOP (69.1%) versus placebo+R‐CHOP (54.9%). The median DOR was not reached in either arm; the 36‐month DOR rate was higher with ibrutinib+R‐CHOP (77.8%) versus placebo+R‐CHOP (64.4%). Similar trends of improvement in EFS (HR = 0.50), PFS (HR = 0.35) and OS (HR = 0.37) were observed with ibrutinib+R‐CHOP treatment in the ABC subpopulation (Online Table SI).

Among patients aged ≥60 years, ibrutinib+R‐CHOP did not improve EFS, PFS, OS or CR rate versus placebo+R‐CHOP (Figures 2D and 3C, Online Table SI).

### 3.4 Safety

The frequency of grade ≥3 treatment‐emergent AEs (TEAEs) was similar between study arms (99 [96·1%] patients in ibrutinib+R‐CHOP versus 94 [97·9%] in placebo+R‐CHOP arm; Table 3). The frequency of grade ≥3 serious TEAEs was higher in ibrutinib+R‐CHOP (47 [45.6%]) versus placebo+R‐CHOP arm (30 [31.3%]). The most common grade ≥3 TEAEs were neutropenia (60.2% vs. 66.7%), white blood cell count decrease (49.5% vs. 57.3%) and leukopenia (35.0% vs. 36.5%). Febrile neutropenia occurred in 21.4% versus 13.5% of patients in the ibrutinib+R‐CHOP versus the placebo+R‐CHOP arm and atrial fibrillation occurred in 3.9% (including 1.0% with grade 3/4) versus 1.0% of patients, respectively.

**TABLE 3:** Treatment‐emergent adverse events in the safety population

	Age <60 years		Age ≥60 years		Total (ITT population – China subgroup)	
	Ibrutinib+	Placebo+	Ibrutinib+	Placebo+	Ibrutinib+	Placebo+
	R‐CHOP	R‐CHOP	R‐CHOP	R‐CHOP	R‐CHOP	R‐CHOP
*n* (%)	*n *= 54	*n *= 51	*n *= 49	*n *= 45	*n *= 103	*n *= 96
TEAEs	54 (100)	51 (100)	49 (100)	45 (100)	103 (100)	96 (100)
Grade ≥3	53 (98·1)	49 (96·1)	46 (93·9)	45 (100·0)	99 (96·1)	94 (97·9)
Study drug‐related	51 (94·4)	46 (90·2)	47 (95·9)	41 (91·1)	98 (95·1)	87 (90·6)
Serious TEAEs	25 (46·3)	15 (29·4)	27 (55·1)	19 (42·2)	52 (50·5)	34 (35·4)
Grade ≥3	23 (42·6)	12 (23·5)	24 (49·0)	18 (40·0)	47 (45·6)	30 (31·3)
Study drug‐related	20 (37·0)	12 (23·5)	24 (49·0)	16 (35·6)	44 (42·7)	28 (29·2)
TEAEs leading to study drug discontinuation	5 (9.3)	6 (11·8)	17 (34·7)	5 (11·1)	22 (21·4)	11 (11·5)
TEAEs leading to death	0	1 (2·0)	1 (2·0)	1 (2·2)	1 (1·0)	2 (2·1)
Serious TEAEs occurring in ≥5% of patients per study group	25 (46·3)	15 (29·4)	27 (55·1)	19 (42·2)	52 (50·5)	34 (35·4)
Febrile neutropenia	9 (16·7)	3 (5·9)	5 (10·2)	5 (11·1)	14 (13.6)	8 (8·3)
Pneumonia	5 (93)	1 (2·0)	5 (10·2)	1 (2·2)	10 (9.7)	2 (2·1)
Lung infection	0	1 (2·0)	7 (14·3)	5 (11·1)	7 (6.8)	6 (6·3)
Interstitial lung disease	3 (5·6)	2 (3·9)	2 (4·1)	2 (4·4)	5 (4.9)	4 (4·2)
Neutropenia	1 (1·9)	2 (3·9)	4 (8·2)	4 (8·9)	5 (4.9)	6 (6·3)
Pneumonitis	3 (5·6)	0	0	1 (2·2)	3 (2.9)	1 (1·0)

Abbreviations: ITT, intent‐to‐treat; TEAE, treatment‐emergent adverse event; R‐CHOP, rituximab plus cyclophosphamide, doxorubicin, vincristine and prednisone.

In patients aged <60 years, the incidence of grade ≥3 AEs (98.1% vs. 96.1%) and AEs leading to treatment discontinuation (9.3% vs. 11.8%) was comparable between the two arms.

In patients aged ≥60 years, the incidence of grade ≥3 AEs was similar in the ibrutinib+R‐CHOP group (93.9%) and the placebo+R‐CHOP group (100%). Serious TEAEs (55.1% vs. 42.2%) and AEs leading to treatment discontinuation (34.7% vs. 11.1%) were more common in patients who received ibrutinib+R‐CHOP than placebo+R‐CHOP.

Twenty‐five (24·0%) patients in the ibrutinib+R‐CHOP arm and 24 (25·0%) in the placebo+R‐CHOP arm discontinued from the study. One patient in the ibrutinib+R‐CHOP arm (multiple organ dysfunction syndrome) and two patients in the placebo+R‐CHOP arm (lung infection and sudden death) had TEAEs leading to death. TEAEs leading to treatment discontinuation of ibrutinib or placebo occurred in 22 (21.4%) patients in the ibrutinib+R‐CHOP arm and 11 (11.5%) patients in the placebo+R‐CHOP arm. Incidence of TEAEs leading to discontinuation of R‐CHOP was higher in patients receiving ibrutinib+R‐CHOP (26·2%) versus placebo+R‐CHOP (15.6%), with the most common being interstitial lung disease (5.5%), lung infection (3.0%) and pneumonitis (2.0%). TEAEs leading to R‐CHOP discontinuation occurred more frequently with ibrutinib+R‐CHOP versus placebo+R‐CHOP in patients aged ≥60 years (36.7% vs. 20.0%) and <60 years (16.7% vs. 11.8%).

### 3.5 Treatment exposure

In the safety population, R‐CHOP exposure was lower for ibrutinib+R‐CHOP (78·6%) than for placebo+R‐CHOP (88.5%) in patients receiving ≥6 cycles of R‐CHOP (Online Table SII). The percentages of patients who had ≥6 cycles of R‐CHOP was similar among those aged <60 years (87.0% vs. 88·2%) and was lower among those aged ≥60 years (67.3% vs. 88.9%), in the ibrutinib+R‐CHOP versus placebo+R‐CHOP arms, respectively.

### 3.6 Biomarker analysis

#### 3.6.1 BCL2/MYC

A total of 182 patients were included in the analyses: 97 in the ibrutinib+R‐CHOP arm and 85 in the placebo+R‐CHOP arm. Eighty patients had *BCL2*‐high/*MYC‐*high co‐expression: 42 (median age, 59.5 years) received ibrutinib+R‐CHOP, 38 (median age, 61.5 years) received placebo+R‐CHOP. Baseline characteristics were balanced, except slightly more patients receiving ibrutinib+R‐CHOP versus placebo+R‐CHOP were aged <60 years (50.0% vs. 39.5%) and female (42.9% vs. 31.6%; Online Table SIII).

**FIGURE 4:**
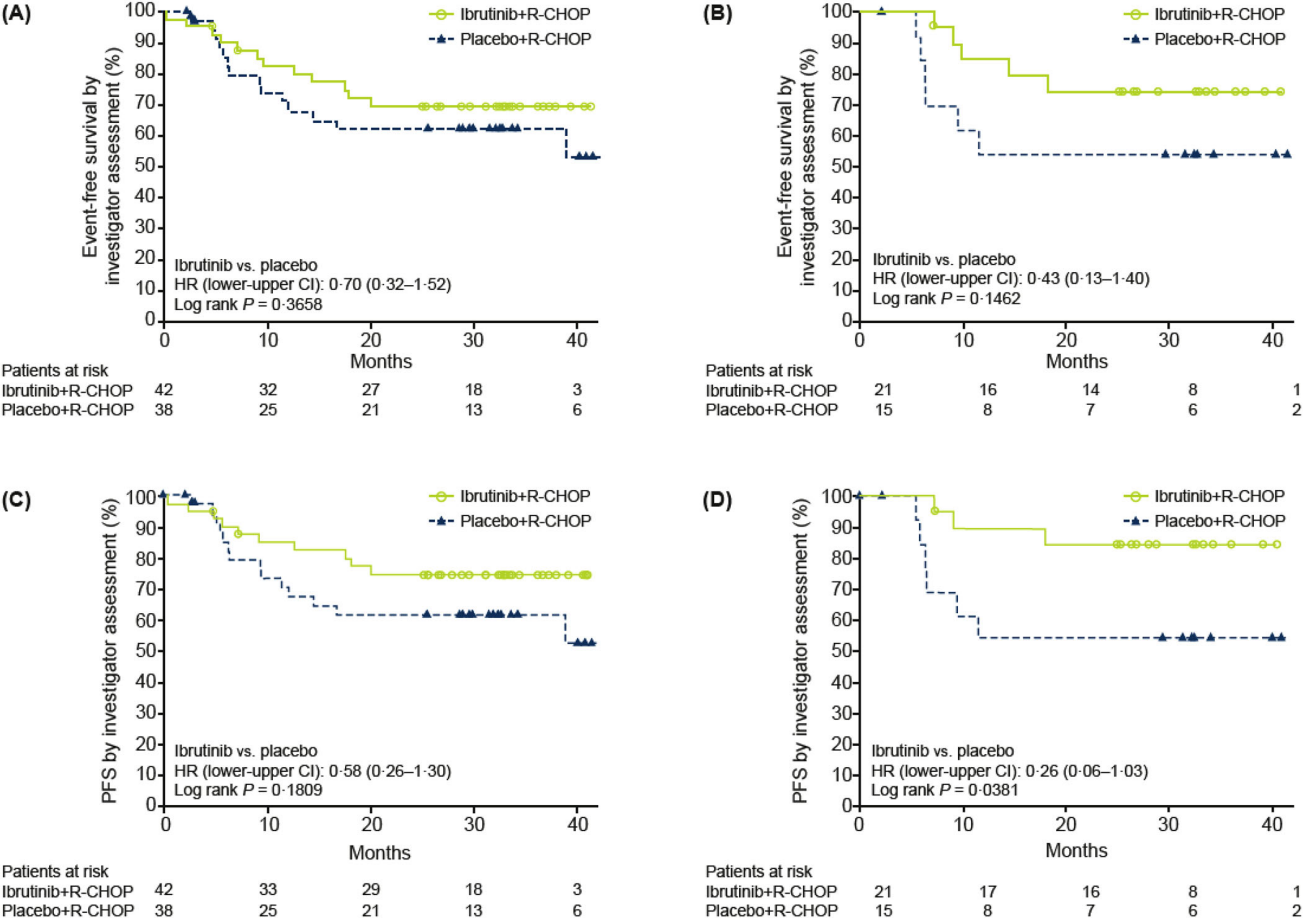
Event‐free survival (EFS) and progression‐free survival (PFS) in patients with *BCL2*‐high/*MYC*‐high co‐expression. (A) EFS in *BCL2*‐high/*MYC*‐high expressors, overall; (B) EFS in *BCL2*‐high/*MYC*‐high expressors, aged <60 years; (C) PFS in *BCL2*‐high/*MYC*‐high expressors, overall; (D) PFS in *BCL2*‐high/*MYC*‐high expressors, aged <60 years. CI, confidence interval; EFS, event‐free survival; HR, hazard ratio; PFS, progression‐free survival; R‐CHOP, rituximab plus cyclophosphamide, doxorubicin, vincristine and prednisone

A numerical trend was seen towards improved EFS (HR = 0.70, lower‐upper CI: 0.32–1.52; *p *= 0.3658) and PFS (HR = 0.58, lower‐upper CI: 0.26–1.30; *p *= 0.1809) with ibrutinib+R‐CHOP versus placebo+R‐CHOP in patients with *MYC*‐high/*BCL2*‐high co‐expression, particularly in patients aged <60 years (EFS: HR = 0.43, lower‐upper CI: 0.13–1.40; *p *= 0.1462; Figure 4). The trend did not meet statistical significance, likely due to the small sample size.

ORR and CR rates were comparable in patients with *MYC*‐high/*BCL2*‐high co‐expression treated with ibrutinib+R‐CHOP versus placebo+R‐CHOP (94.9% vs. 94.6% and 64.1% vs. 59.5%, respectively).

### 3.7 Whole exome analysis

One hundred one patients in the ibrutinib+R‐CHOP arm and 88 patients in the placebo+R‐CHOP arm were included in the analysis. The frequency of mutations (single‐nucleotide variants and indels) was 23.3% for *CD79B*, 15.3% for *MYD88* L265P, 7.4% for *TP53*, 6.4% for *CARD11_CC* and 5.8% for both *MYD88* L265P and *CD79B*. Of patients with *TP53* mutations, 10 of 12 (83.3%) in the ibrutinib+R‐CHOP arm and 1 of 2 (50.0%) in the placebo+R‐CHOP arm had a CR (Table 4, Online Table SIV). Whole exome sequencing analyses showed that mutations in *PARK2* were associated with improvements in EFS (HR = 0.05, 95% CI: 0.01–0–42; *p *= 0.0002) and OS (HR = 0.05, 95% CI: 0.01–0.44; *p *= 0.0002) with ibrutinib+R‐CHOP versus placebo+R‐CHOP treatment (Online Tables S5 and S6, Online Figure S1).

**TABLE 4:** Percentage of patients with selected gene mutations among patients with CR or non‐CR by treatment arm

	Ibrutinib+R‐CHOP		Placebo+R‐CHOP		OR (95% CI) *p* value Ibrutinib+R‐CHOP versus placebo+R‐CHOP	
Gene mutations/combination	CR (*n* = 66)	Non‐CR (*n* = 29)	CR (*n* = 50)	Non‐CR (*n* = 34)	CR	Non‐CR
*TP53*, *n* (%)	10 (15·2)	1 (3·4)	1 (2·0)	1 (2·9)	8·630 (1·154–386·618) *p* = 0·023	1·175 (0·015–95·209) *p* = 1·000
*MYD88_L265P* not *CD79B, n* (%)	4 (6·1)	2 (6·9)	7 (14·0)	3 (8·8)	0·400 (0·081–1·686) *p* = 0·203	0.769 (0·060–7·239) *p* = 1·000
*CD79B, n* (%)	14 (21·2)	6 (20·7)	16 (32·0)	5 (14·7)	0·575 (0·227–1·439) *p* = 0·206	1·503 (0·334–7·086) *p* = 0·741
*MYD88_L265P*, *n* (%)	9 (13·6)	3 (10·3)	12 (24·0)	3 (8·8)	0·503 (0·169–1·446) *p* = 0·223	1·189 (0·146–9·655) *p* = 1·000
*CD79B* not *MYD88_L265P*, *n* (%)	9 (13·6)	5 (17·2)	11 (22·0)	5 (14·7)	0·563 (0·187–1·653) *p* = 0·321	1.205 (0·245–5·922) *p* = 1·000
*MYD88_L265P* and *CD79B*, *n* (%)	5 (7·6)	1 (3·4)	5 (10·0)	0	0·740 (0·160–3·422) *p* = 0·743	Inf (0·030‐Inf) *p* = 0·460

*Note*: Non‐CR is defined as partial response + SD + PD.

Abbreviations: CI, confidence interval; CR, complete response; OR, odds ratio; PD, progressive disease; R‐CHOP, rituximab plus cyclophosphamide, doxorubicin, vincristine and prednisone; SD; stable disease.

## 4 DISCUSSION

In the Chinese subpopulation from the PHOENIX trial, ibrutinib+R‐CHOP did not improve EFS versus placebo+R‐CHOP for the ITT or ABC populations. However, in Chinese patients aged <60 years, ibrutinib+R‐CHOP provided benefit versus placebo+R‐CHOP for EFS, PFS and CR rate, with manageable safety. The DOR of patients who achieved response was longer, and a numerical trend of improved OS with ibrutinib+R‐CHOP was also seen in Chinese patients aged <60 years. In Chinese patients ≥60 years, ibrutinib+R‐CHOP was associated with increased toxicity, leading to higher rates of R‐CHOP discontinuation and inferior outcomes versus placebo+R‐CHOP treatment. Rates of serious TEAEs were higher in the ibrutinib+R‐CHOP arm in both age subgroups; however, among patients aged <60 years, the number receiving ≥6 cycles of R‐CHOP was comparable between treatment arms. In contrast, among patients aged ≥60 years, a lower proportion in the ibrutinib+R‐CHOP arm received ≥6 cycles of R‐CHOP. Decreased tolerance to ibrutinib treatment in patients aged ≥60 years led to reduced treatment exposure, likely explaining the lower efficacy observed in this subgroup. The concordance between immunohistochemistry enrichment for non‐GCB DLBCL and GEP classification was 84.3% (150/178) of the patients with valid GEP data, among whom the ABC subtype was identified in 79.2% (141/178). This result aligns with previously published concordance rates of approximately 80% between immunohistochemistry and GEP classification methods [19]. The efficacy and safety outcomes in the Chinese subgroup are consistent with those in the overall study population [18].

Baseline demographics in the Chinese subgroup matched well with the total ITT population, except with a slightly younger median age of 59 years versus 62 years for the total ITT population, in line with a younger DLBCL patient population in China [18]. In several epidemiological studies in China, the median age of patients with DLBCL was 55–57 years, about 10 years younger than Western patients [2, 20, 21, 22]. Additionally, this study population included a higher percentage of men than women, reflecting the higher rate of diagnosed DLBCL in men than women (1.16:1) in China [22] and the higher age‐standardised incidence rate of NHL in China for males versus females in all age groups, with a two‐fold greater risk seen for males versus females in the 55–59‐year age group [2].

R‐CHOP has represented the standard first‐line treatment for DLBCL for 20 years and is also the standard first‐line treatment for DLBCL in China. Real‐world evidence has shown R‐CHOP to provide significant OS and PFS benefit over CHOP in Chinese patients aged ≥60 years and in those with non‐GCB‐subtype DLBCL [8, 9]; however, worse survival outcomes have been associated with high *MYC*/*BCL2* co‐expression in Chinese patients with DLBCL treated with R‐CHOP [15]. Additionally, some previous findings have suggested that *BCL2* overexpression might be more common in Chinese versus Western patients with DLBCL [5]. In this subgroup analysis of Chinese patients in the PHOENIX study, R‐CHOP efficacy was consistent with previous clinical study results and real‐world data in Chinese patients with DLBCL [10, 11, 12, 13]. In addition, ibrutinib+R‐CHOP significantly improved PFS in Chinese patients aged <60 years with *BCL2*‐high/*MYC*‐high co‐expression. Similar trends in EFS, PFS and OS were also observed in patients aged <60 years in the ABC subpopulation, although these did not reach statistical significance.

Even though the number of patients in the biomarker analyses is low, results showed that patients with *TP53* mutations were more likely to achieve CR with ibrutinib+R‐CHOP versus placebo+R‐CHOP. Notably, *TP53* mutations have previously been found to predict poor survival in CHOP‐treated patients with GCB DLBCL and in R‐CHOP‐treated patients with either GCB or ABC DLBCL [23, 24]. While both *TP53* and NF‐κB transcription factors respond to different types of stress, mutant p53 proteins have been reported to enhance the induction of NF‐κB activity [25]. In this analysis, it is possible that patients with mutated *TP53* have an activated NF‐κB pathway and upregulated Bruton's tyrosine kinase, which in turn might lead to better outcomes with ibrutinib+R‐CHOP.

Our results also showed that *PARK2* mutations were associated with better EFS and OS with ibrutinib+R‐CHOP versus placebo+R‐CHOP. Alterations in *PARK2* are highly common in human cancers, and the gene product of *PARK2*, the ubiquitin E3 ligase Parkin, has been shown to inhibit both NF‐κB and PI3K/AKT signalling and is a key activator for the cell death pathway induced by TNFR1 and its ligands [26, 27, 28]. The difference between the results for wild‐type and mutated *PARK2* in this study was seen in the response to placebo+R‐CHOP rather than ibrutinib+R‐CHOP, suggesting that in wild‐type *PARK2*, chemotherapy can induce apoptosis via the death pathway, whereas this response is inhibited in *PARK2*‐mutated tumours, making them more resistant to chemotherapy. As there were a limited number of patients, these biomarker data should be interpreted with caution. Further understanding of how ibrutinib regulates the immune micro‐environment and determination of biological and molecular signatures for potential identification of certain subgroups of non‐GCB Chinese patients who might benefit from an ibrutinib‐based regimen might provide directions for future clinical study design and clinical practice.

## 5 CONCLUSIONS

Given the highly heterogeneous nature of DLBCL and lack of an effective standard of care for younger patients with DLBCL in China, the results of this subgroup analysis of Chinese patients enrolled in the PHOENIX study aligned with the encouraging trend observed in patients aged <60 years in the overall population. Further, our biomarker data are hypothesis generating for potential identification of certain subgroups of Chinese patients with non‐GCB DLBCL who might experience improved response with ibrutinib+R‐CHOP versus placebo+R‐CHOP treatment.

## CONFLICT OF INTEREST

S W, S M S, S S, Y Z, and J V: Janssen employment and stock ownership from Johnson and Johnson; B H, Z W, X W and Y F: Janssen employment; J Z, X H, Y S, S B, Q Z, Y S, H H, H Z and WW have no conflict of interest to disclose.

## AUTHOR CONTRIBUTIONS

J Z, X H, B H, S B, S W, S S, W W and J V provided research and analysis of the concept and design; J Z provided financial and administrative support; J Z, X H, Y Q S, Q Z, Y S, H H, H Z, S M S and J V provided treatment, follow‐up, monitoring, data collection and assembly; J Z, X H, Y Q S, B H, S B, S W, Q Z, Y S, H H, H Z, S M S, S S, Y Z, Y F, W W and J V performed research and analysis. All authors contributed to the writing of the manuscript, approved the final version and were accountable for all aspects of the work.

## Supporting information



Supporting Information

## Data Availability

The data sharing policy of the Janssen Pharmaceutical Companies of Johnson and Johnson is available at www.janssen.com/clinical‐trials/transparency. Requests for access to data from select studies can be submitted through the Yale Open Data Access (YODA) Project site at yoda.yale.edu
