# Supplementary material for: Ibrutinib and rituximab plus cyclophosphamide, doxorubicin, vincristine and prednisone in patients with previously untreated non‐germinal centre B‐cell‐like diffuse large B‐cell lymphoma: A Chinese subgroup analysis of the phase III PHOENIX trial
Source: EJHaem. 2022 Aug 30;3(4):1154–64. doi: 10.1002/jha2.517 (PMC9713042; doi:10.1002/jha2.517)
Supplement: Supplementary file 1 — Supporting Information [file JHA2-3-1154-s001.docx]

**Supplementary Appendix**

**Ethical Approvement Statment**

The study was approved by the institutional review board or independent ethics committee at each participating institution and conducted in accordance with ethical principles defined by the Declaration of Helsinki and the International Conference on Harmonisation Guidelines for Good Clinical Practice. An independent data monitoring committee reviewed safety and risk/benefit. All patients provided informed consent.

**Concordance and biomarker analysis**

Diffuse large B-cell lymphoma (DLBLC) subtype was by Hans-based immunohistochemistry. Retrospective subtyping of available tumour tissues was performed via gene expression profiling (GEP) methods using the HTG EdgeSeq DLBCL Cell-of-Origin assay; concordance between immunohistochemistry and GEP classification of molecular subtype was determined using matching tumour or blood samples.

Pre-planned biomarker analyses included evaluation of *BCL2* and *MYC* gene expression by RNA-seq. Patients with above median transcript per million expression values of both *BCL2* and *MYC* in the intent-to-treat (ITT) population were classified as “double expressors” and differences in time to event factors were evaluated in this population.

Whole exome sequencing, approved by Human Genetic Resources Administration of China, was conducted on formalin-fixed, paraffin-embedded tumour samples, with a focus on genes that were previously investigated in relation to ibrutinib activity in DLBCL, including *MYD88*, *TP53*, *CARD11* and *CD79B*. Variant frequencies was assessed for each gene and for several combinations of genes by treatment outcomes.

Kaplan-Meier analysis was used to correlate treatments with efficacy (EFS, PFS, OS) within *BCL2/MYC* double-expressor patients. Associations between genomic features and efficacy endpoints were examined using Fisher’s exact test for binary variables or the log-rank test and Cox proportional hazards model for time to event variables, as appropriate.

**Supplementary Table SI.** Efficacy in Chinese patients with ABC-subtype DLBCL.

|  | Age <60 years | | Age ≥60 years | | Total (ABC population –  China subgroup) | |
| --- | --- | --- | --- | --- | --- | --- |
|  | Ibrutinib+  R-CHOP  *n* = 34 | Placebo+ R-CHOP  *n* = 34 | Ibrutinib+ R-CHOP  *n* = 38 | Placebo+ R-CHOP  *n* = 35 | Ibrutinib+ R-CHOP  *n* = 72 | Placebo+ R-CHOP  *n* = 69 |
| **EFS** |  |  |  |  |  |  |
| No. of events, *n* (%) | 7 (20·6) | 12 (35·3) | 13 (34·2) | 10 (28·6) | 20 (27·8) | 22 (31·9) |
| HR (95% CI)  *P* value | 0·498 (0·196–1·267)  0.1358 | | 1·309 (0·573–2·990)  0·5222 | | 0·857 (0·467–1·570)  0·6160 | |
| Median (95% CI), months | NE  (NE-NE) | NE  (13·63–NE) | NE  (14·09–NE) | NE  (38·67–NE) | NE  (NE–NE) | NE  (38·67–NE) |
| 36-month EFS rate, % (95% CI) | 77.0  (57·7–88·3) | 60.5  (41·0–75·4) | 64.0  (46·2–77·3) | 72.7  (54·1–84·8) | 70.0  (57·5–79·5) | 66.8  (53·7–76·9) |
| **PFS** |  |  |  |  |  |  |
| No. of events, *n* (%) | 5 (14·7) | 12 (35·3) | 13 (34·2) | 10 (28·6) | 18 (25·0) | 22 (31·9) |
| HR (95% CI)  *P* value | 0·352 (0·124–1·001)  0·0405 | | 1·309 (0·573–2·990)  0·5222 | | 0·764 (0·410–1·425)  0·3958 | |
| Median (95% CI), months | NE  (NE–NE) | NE  (13·63–NE) | NE  (14·09–NE) | NE  (38·67–NE) | NE  (NE–NE) | NE  (38·67–NE) |
| 36-month PFS rate, % (95% CI) | 83·7  (65·1–92·9) | 60·5  (41·0–75·4) | 64·0  (46·2–77·3) | 72·7  (54·1–84·8) | 73·1  (60·7–82·1) | 66·8  (53·7–76·9) |
| **OS** |  |  |  |  |  |  |
| No. of events, *n* (%) | 3 (8·8) | 7 (20·6) | 10 (26·3) | 6 (17·1) | 13 (18·1) | 13 (18·8) |
| HR (95% CI)  *P* value | 0·373 (0·096–1·444)  0·1372 | | 1·615 (0·587–4·444)  0·3489 | | 0·928 (0·430–2·002)  0·8488 | |
| Median (95% CI), months | NE (NE–NE) | NE (NE–NE) | NE (NE–NE) | NE (NE–NE) | NE (NE–NE) | NE (NE–NE) |
| 36-month OS rate, % (95% CI) | 90·7  (73·9–96·9) | 77·0  (57·8–88·3) | 72·4  (54·7–84·1) | 82·5  (65·1–91·7) | 81·1  (69·6–88·6) | 80·0  (68·1–87·9) |
| **Best response, n (%)** |  |  |  |  |  |  |
| Overall response | 32 (94·1) | 28 (82·4) | 31 (81·6) | 33 (94·3) | 63 (87·5) | 61 (88·4) |
| Complete response | 24 (70·6) | 16 (47·1) | 24 (63·2) | 25 (71·4) | 48 (66·7) | 41 (59·4) |
| Partial response | 8 (23·5) | 12 (35·3) | 7 (18·4) | 8 (22·9) | 15 (20·8) | 20 (29·0) |

ABC, activated B cell-like; CI, confidence interval; DLBCL, diffuse large B-cell lymphoma; EFS, event-free survival; HR, hazard ratio; ITT, intent-to-treat; NE, not evaluable; OS, overall survival; PFS, progression-free survival; R-CHOP, rituximab plus cyclophosphamide, doxorubicin, vincristine and prednisone.

**Supplementary Table SII.** Extent of drug exposure by age.

| *n* (%) | ITT population – China subgroup | | Age <60 years | | Age ≥60 years | |  |
| --- | --- | --- | --- | --- | --- | --- | --- |
| No. of cycles received | Ibrutinib+  R-CHOP  (*n* = 103) | Placebo+  R-CHOP (*n* = 96) | Ibrutinib+  R-CHOP  (*n* = 54) | Placebo+  R-CHOP (*n* = 51) | Ibrutinib+  R-CHOP (*n* = 49) | Placebo+  R-CHOP (*n* = 45) |  |
| Ibrutinib/placebo | | | | | | | |
| <6 | 22 (21·4) | 11 (11·5) | 6 (11·1) | 6 (11·8) | 16 (32·7) | 5 (11·1) |  |
| ≥6 | 81 (78·6) | 85 (88·5) | 48 (88·9) | 45 (88·2) | 33 (67·3) | 40 (88·9) |  |
| R-CHOP (any one or more components) | | | | | | | |
| <6 | 23 (22·3) | 11 (11·5) | 7 (13·0) | 6 (11·8) | 16 (32·7) | 5 (11·1) |  |
| ≥6 | 80 (77·7) | 85 (88·5) | 47 (87·0) | 45 (88·2) | 33 (67·3) | 40 (88·9) |  |

R-CHOP, rituximab + cyclophosphamide, doxorubicin, vincristine and prednisone.

**Supplementary Table SIII.** Baseline characteristics of Chinese patients with *BCL2*-high/*MYC*-high expression in the phase III PHOENIX study.

|  | Ibrutinib+ R-CHOP  *n* = 42 | Placebo+ R-CHOP  *n* = 38 |
| --- | --- | --- |
| Age, years  Mean (SD)  Median (range) | 59·9 (7·90)  59·5 (33–75) | 55·2 (16·21)  61·5 (19–78) |
| Age groups, *n* (%)  <60 years  ≥60 years | 21 (50·0)  21 (50·0) | 15 (39·5)  23 (60·5) |
| Sex, *n* (%)  Female  Male | 18 (42·9)  24 (57·1) | 12 (31·6)  26 (68·4) |
| Time from initial diagnosis to randomisation, days  Mean (SD)  Median (range) | 30·3 (44·12)  20·0 (6–302) | 25·4 (14·72)  20·0 (9–74) |
| Baseline stage of DLBCL at entry, *n* (%)  I  II  III  IV | 0  12 (28·6)  14 (33·3)  16 (38·1) | 0  18 (47·4)  8 (21·1)  12 (31·6) |
| Baseline lymphoma symptoms, *n* (%) | 8 (19·0) | 9 (23·7) |
| Bone marrow involvement, *n* (%)* | 1 (2·4) | 0 |
| ECOG PS, *n* (%)  0  1  2 | 13 (31·0)  24 (57·1)  5 (11·9) | 12 (31·6)  16 (42·1)  10 (26·3) |
| Bulky tumour (long axis ≥10 cm), *n* (%) | 3 (7·1) | 4 (10·5) |
| No. of extranodal sites, n (%)  0  1  >1 | 13 (31·0)  16 (38·1)  13 (31·0) | 17 (44·7)  10 (26·3)  11 (28·9) |
| IPI/R-IPI score index number, *n* (%)  0  1  2  3  4  5 | 0  11 (26·2)  14 (33·3)  15 (35·7)  2 (4·8)  0 | 0  15 (39·5)  9 (23·7)  9 (23·7)  4 (10·5)  1 (2·6) |
| Elevated LDH, n (%) | 23 (54·8) | 17 (44·7) |
| No. of planned treatment cycles (used in stratification), *n* (%)  6  8 | 7 (16·7)  35 (83·3) | 9 (23·7)  29 (76·3) |
| GEP^†^ subtype, *n* (%)  ABC  Unclassified  GCB  Unknown | 35 (83·3)  2 (4·8)  2 (4·8)  3 (7·1) | 32 (84·2)  0  5 (13·2)  1 (2·6) |

*Defined as any baseline aspirate or biopsy result of histology positive or histology negative/intermediate that is confirmed positive by immunohistochemistry or flow cytometry; ^†^Conducted after non-GCB enrichment by immunohistochemistry.

ABC, activated B cell-like; DLBCL, diffuse large B-cell lymphoma; ECOG PS, Eastern Cooperative Oncology Group performance status; GCB, germinal centre B cell-like; GEP, gene expression profiling; IPI, International Prognostic Index; LDH, lactate dehydrogenase; R-CHOP, rituximab + cyclophosphamide, doxorubicin, vincristine, and prednisone; R-IPI: revised International Prognostic Index; SD, standard deviation.

**Supplementary Table SIV.** Overall response rate in patients with common gene mutations.

| Gene mutation | Treatment | ORR, *n/N* (%) | CR, *n/N* (%) | PR, *n/N* (%) | SD (%) | PD, *n/N* (%) | NE, *n/N* (%) |
| --- | --- | --- | --- | --- | --- | --- | --- |
| *TP53* | Ibrutinib+ R-CHOP | 11/12 (91·7) | 10/12 (83·3) | 1/12 (8·3) | 0 | 1/12 (8·3) | 0 |
|  | Placebo+  R-CHOP | 2/2 (100) | 1/2 (50·0) | 1/2 (50·0) | 0 | 0 | 0 |
| *CD79B* | Ibrutinib+  R-CHOP | 18/20 (90·0) | 14/20 (70·0) | 4/20 (20·0) | 0 | 2/20 (10·0) | 0 |
|  | Placebo+  R-CHOP | 20/22 (90·9) | 16/22 (72·7) | 4/22 (18·2) | 0 | 1/22 (4·5) | 1/22 (4·5) |
| *MYD88_L265P* | Ibrutinib+ R-CHOP | 12/12 (100) | 9/12 (75·0) | 3/12 (25·0) | 0 | 0 | 0 |
|  | Placebo+  R-CHOP | 14/15 (93·3) | 12/15 (80·0) | 2/15 (13·3) | 0 | 1/15 (6·7) | 0 |
| *MYD88_L265P* not *CD79B* | Ibrutinib+  R-CHOP | 6/6 (100) | 4/6 (66·7) | 2/6 (33·3) | 0 | 0 | 0 |
|  | Placebo+  R-CHOP | 9/10 (90·0) | 7/10 (70·0) | 2/10 (20·0) | 0 | 1/10 (10·0) | 0 |
| *MYD88_L265P* and *CD79B* | Ibrutinib+  R-CHOP | 6/6 (100) | 5/6 (83·3) | 1/6 (16·7) | 0 | 0 | 0 |
|  | Placebo+  R-CHOP | 5/5 (100) | 5/5 (100·0) | 0 | 0 | 0 | 0 |

CR, complete response; NE, not evaluable; ORR, overall response rate; PD, progressive disease; PR, partial response; R-CHOP, rituximab + cyclophosphamide, doxorubicin, vincristine and prednisone; SD, stable disease.

**Supplementary Table SV.** EFS by treatment arm in patients with top 10 gene mutations, selected by smallest log-rank nominal *P* values.

| Gene mutation | Treatment | Event/Total (%) | Median EFS (95% CI) | HR (95% CI) | *P* value (log rank) |
| --- | --- | --- | --- | --- | --- |
| *PARK2* | Placebo+  R-CHOP | 5/5 (100·0) | 5·8 (2·1–NE) | 0·05 (0·01-0·42) | 0·0002 |
|  | Ibrutinib+  R-CHOP | 3/10 (30·0) | 43·6 (NE–NE) |  |  |
| *RYR1* | Placebo+ R-CHOP | 3/4 (75·0) | 6.3 (5·3–NE) | 0·00 (0·00-Inf) | 0·0008 |
|  | Ibrutinib+  R-CHOP | 2/7 (28·6) | NE (18·1–NE) |  |  |
| *AFF1* | Placebo+ R-CHOP | 7/9 (77·8) | 13.6 (6·3–NE) | 0·12 (0·0-0·59) | 0·0019 |
|  | Ibrutinib+  R-CHOP | 2/13 (15·4) | NE (NE–NE) |  |  |
| *LAMC2* | Placebo+ R-CHOP | 4/4 (100·0) | 7·8 (4·6–NE) | 0·06 (0·01-0·60) | 0·0020 |
|  | Ibrutinib+  R-CHOP | 1/7 (14·3) | NE (NE–NE) |  |  |
| *NRP1* | Placebo+ R-CHOP | 4/4 (100·0) | 10·3 (51–NE) | 0·07 (0·01-0·63) | 0·0022 |
|  | Ibrutinib+  R-CHOP | 1/8 (12·5) | NE (NE–NE) |  |  |
| *PRDM1* | Placebo+ R-CHOP | 9/16 (56·3) | 19·1 (9·4–NE) | 0·13 (0·03-0·62) | 0·0026 |
|  | Ibrutinib+  R-CHOP | 2/19 (10·5) | NE (NE–NE) |  |  |
| *RSBN1L* | Placebo+  R-CHOP | 4/4 (100) | 5·4 (5·3–NE) | 0·00 (0·00-Inf) | 0·0027 |
|  | Ibrutinib+ R-CHOP | 0/5 (0) | NE (NE–NE) |  |  |
| *BRD7* | Placebo+ R-CHOP | 4/8 (50·0) | 9·5 (5·8–NE) | 0·00 (0·00-Inf) | 0·0028 |
|  | Ibrutinib+  R-CHOP | 0/12 (0·0) | NE (NE–NE) |  |  |
| *GRM7* | Placebo+ R-CHOP | 2/2 (10·.0) | 7.8 (5·5–NE) | 0·00 (0·00–Inf) | 0·0039 |
|  | Ibrutinib+  R-CHOP | 0/7 (0·0) | NE (NE–NE) |  |  |
| *ZNF432* | Placebo+  R-CHOP | 1/6 (16·7) | NE (NE–NE) | 1·23E10 (0·00–Inf) | 0·0039 |
|  | Ibrutinib+  R-CHOP | 2/2 (100·0) | 9·0 (8·3–NE) |  |  |

CI, confidence interval; EFS, event-free survival; NE, not estimable; HR, hazard ratio; R-CHOP, rituximab + cyclophosphamide, doxorubicin, vincristine and prednisone.

**Supplementary Table SVI.** OS by treatment arm in patients with top 10 gene mutations, selected by smallest log-rank nominal *P* values.

| Gene mutation | Treatment | Event/Total (%) | Median OS (95% CI), months | HR (95% CI) | *P* value (log rank) |
| --- | --- | --- | --- | --- | --- |
| *PARK2* | Placebo+  R-CHOP | 5/5 (100·0) | 5·8 (2·1–NE) | 0·05 (0·01–0·44) | 0·0002 |
|  | Ibrutinib+  R-CHOP | 2/10 (20·0) | NE (NE–NE) |  |  |
| *DNTTIP2* | Placebo+ R-CHOP | 0/12 (0·0) | NE (NE-NE) | 6·54E09 (0·00–Inf) | 0·0017 |
|  | Ibrutinib+ R-CHOP | 4/6 (66·7) | 22·7 (9·3–NE) |  |  |
| *HN1L* | Placebo+ R-CHOP | 1/8 (12·5) | NE (NE–NE) | 15·57 (1·56–155·49) | 0·0023 |
|  | Ibrutinib+ R-CHOP | 3/3 (100·0) | 4·6 (2·8–NE) |  |  |
| *TCEB3B* | Placebo+  R-CHOP | 2/2 (100·0) | 11·5 (4·6–NE) | 0·00 (0·00–Inf) | 0·0039 |
|  | Ibrutinib+ R-CHOP | 1/6 (16·7) | NE (NE–NE) |  |  |
| *LRRTM2* | Placebo+  R-CHOP | 3/4 (75·0) | 10·8 (10·0–NE) | 0·00 (0·00–Inf) | 0·0042 |
|  | Ibrutinib+  R-CHOP | 0/5 (0·0) | NE (N–-NE) |  |  |
| *ATAD5* | Placebo+ R-CHOP | 1/1 (100·0) | 2·1 (NE–NE) | 0·00 (0·00–Inf) | 0·0047 |
|  | Ibrutinib+  R-CHOP | 2/8 (25·0) | NE (18·2–NE) |  |  |
| *RAPGEF2* | Placebo+  R-CHOP | 0/12 (0·0) | NE (NE–NE) | 2·23E09 (0·00–Inf) | 0·0049 |
|  | Ibrutinib+  R-CHOP | 6/11 (54·5) | 29·9 (7·2–NE) |  |  |
| *DDX60* | Placebo+ R-CHOP | 2/3 (66·7) | 12·1 (11·4–NE) | 0·00 (0·00–Inf) | 0·0068 |
|  | Ibrutinib+  R-CHOP | 0/9 (0·0) | NE (NE–NE) |  |  |
| *B4GALNT4* | Placebo+ R-CHOP | 2/2 (100·0) | 6·4 (2·1–NE) | 0·00 (0·00–Inf) | 0·0082 |
|  | Ibrutinib+  R-CHOP | 1/5 (20·0) | NE (20·5–NE) |  |  |
| *CDH13* | Placebo+ R-CHOP | 2/2 (100·0) | 14·6 (10·8–NE) | 0·00 (0·00–Inf) | 0·0082 |
|  | Ibrutinib+  R-CHOP | 0/5 (0·0) | NE (NE–NE) |  |  |

CI, confidence interval; HR, hazard ratio; MUT, mutant; NE, not estimable; R-CHOP, rituximab + cyclophosphamide, doxorubicin, vincristine and prednisone.

**Supplementary Fig S1.** Survival in patients with *PARK2* mutations. (A) EFS in patients with *PARK2* mutations; (B) OS in patients with *PARK2* mutations.

EFS, event-free survival; OS, overall survival.


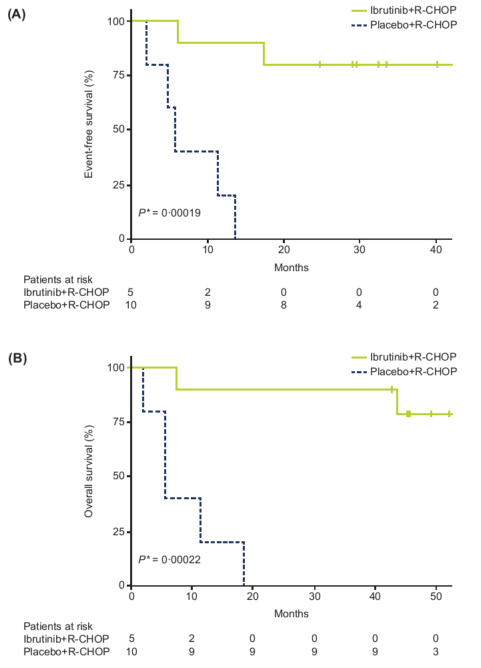


*All *P* values are nominal.
